# Is the White Blood Corpuscle and the Pus Corpuscle Identical?

**Published:** 1875-04

**Authors:** T. Curtis Smith

**Affiliations:** Middleport Ohio


### IS THE WHITE-BLOOD CORPUSCLE AND THE PUS CORPUSCLE IDENTICAL?

BY T. CURTIS SMITH, M. D., MIDDLEPORT, OHIO.

Are pus and white-blood corpuscles identical? Conhiem was, I believe, first to declare their identity, and his statements have been and still are, by very many, accepted as correct. That they resemble each other in appearance is undoubtedly true, and from this resemblance, no doubt, came the theory of their being one and the same. From this, also springs the doctrine that pus was but a secretion from the blood, effected through a degenerative inflammatory process; and some compared its extraction from the blood to be similar, in method, to the extraction of bile or urine.

What pus is, we shall probably not attempt to prove; but we propose to offer a few reasons for the belief that it is a widely different fluid from the white-blood corpuscles. Their resemblance is almost or quite identical in nearly all respects; but we must not forget that mere resemblance is far from proof of actual identity. Two substances may present the same appearance, and yet be widely different in the power or influence they may exert on the animal economy.

The white-blood corpuscle may have but a single nucleus, but it more frequently, or nearly always, has several. If I am correctly informed, the pus corpuscle always has more than one nucleus.—The white-blood corpuscles are found in the blood in the proportion, nominally, of one to three hundred corpuscles. These are distributed throughout all of the circulating fluid. Pus globules are found within circumscribed limits, and often hemmed within those limits by a pyogenic membrane, which prevents their absorption and their being carried into the circulation. Here there is a distinction as to the manner in which they are treated by the system. But this is not all. Pus as pus is not absorbed, and is never found floating in the blood, except in those instances where a vein or lymphatic has sufficiently suffered from an ulcerative process to admit of the entrance of the pus globules as such. The degeneration of pus may, and does, permit the absorption of its watery portion, which is always so abundant, and from this septic symptoms may arise. When such is the case, we find an abscess filled with what we often call cheesy matter, abounding in a superabundance of fatty matter, etc., the thin aqueous portion having been taken up by absorption. A point of resemblance between two kinds of corpuscles is observed in inflammatory action; for during this process many of the white-blood corpuscles pass through the walls of the capillaries in the inflamed portion and its vicinity. These continue their amœbiform movements, which movement is the same as that possessed by the pus corpuscle when fresh. Pus occurs as the result of inflammation, and where inflammation exists, there are large numbers of the "wandering cells," or white corpuscles. "Conhiem concluded, therefore, that pus corpuscles came from the white corpuscles, or 'wandering cells,' and as the 'wandering cells' came from the white-blood corpuscles, therefore, that a pus corpuscle was a white-blood corpuscle," and rejected the teachings that pus could be derived from any other source. This conclusion has been very widely accepted. This is a plausible theory, but will it bear the test of experience? I think the proof of their identity is wanting in more points than one. As stated before, mere similarity of appearance, even in every particular, is not proof positive of their identity. The white corpuscles are only representatives of one stage of their development, having no well-known function, as have the red corpuscles. This being the case, it is not hard to see how they can undergo a retrograde metamorphosis outside of the vessels, very easily, and especially where the heat and activity of the circulation is abnormally increased. But because they undergo such retrograde change, and become pus corpuscles, does not make them oue and the same thing. Besides, proof is wanting that such transformation is the only source of the formation of pus.

The white corpuscles of the blood of different persons resemble each other, and their properties are so nearly identical that, by transfusion, those of one person can be substituted for those of another without serious disturbance to the person receiving the transfused blood. Now, if the pus corpuscle is identical with the white-blood corpuscle, there should be no danger in introducing them into the system. Is such tho case? By no means. It has been clearly proven that the introduction of pus into the system, both men and animals, has led to serious consequences, and to a fatal disease known as pyemia. Thus if pus, be it ever so laudable, be introduced into the circulation of a dog, serious systemic disturbance is at once set up, and death often ıesults. If some of the blood from this dog be injected into the veins of another dog, still more serious results attain than in the first instance, and so on. Another proof of the non-identity of these globules is the fact that pus itself differs in its properties. Thus, pus from the gonorrheal urethra is by no means the same as pus from a chancre; nor is pus from either of these the same as that found in a variolus pustule; and each of these possess properties quite different from that of phlegmonous erysipelas, and from the pus of an ordinary furuncle. These great differences certainly should bear some weight in the argument against the identity of the pus and white-blood corpuscle.

Another wide and distinctive difference between the two is the fact that the white-blood corpuscle is capable of becoming organized; and it is a well-known fact that the new formations resulting from the inflammatory process are largely the result of the organization of these white wandering cells, and some have claimed that all new growths of connective tissue and new formations result from these.

We do know beyond the possibility of dispute that pus never can become organized, and that, once formed, it will remain pus while it is retained in any part of the system, or until it becomes degenerated—destroyed. So that, even if pus originates from the white or wandering cells, it has at least lost its vital power. But I think experience has proven to us that pus is also formed from the destruction of tissue and from the red as well as the white blood, for in cases of long continuance and profuse suppuration we cannot avoid observing the very pale and anemic condition of the patient, and the fact also that the blood of such patients is not only deficient in globuline and hæmatine, but deficient also in plasma. From these causes, nutrition is but imperfectly carried on, and, in time, emaciation and great general debility result as a consequence.

